# Pharmacological Efficacy of Probiotics in Respiratory Viral Infections: A Comprehensive Review

**DOI:** 10.3390/jpm12081292

**Published:** 2022-08-06

**Authors:** Shadma Wahab, Dalia Almaghaslah, Syed Esam Mahmood, Md Faruque Ahmad, Abdulrahman A. Alsayegh, Yahya M. Abu Haddash, Mohammad Akhlaquer Rahman, Irfan Ahamd, Wasim Ahmad, Mohammad Khalid, Shazia Usmani, Md Parwez Ahmad, Umme Hani

**Affiliations:** 1 Department of Pharmacognosy, College of Pharmacy, King Khalid University, Abha 61421, Saudi Arabia; 2 Department of Clinical Pharmacy, College of Pharmacy, King Khalid University, Abha 61421, Saudi Arabia; 3 Department of Family and Community Medicine, College of Medicine, King Khalid University, Abha 61421, Saudi Arabia; 4 Department of Clinical Nutrition, College of Applied Medical Sciences, Jazan University, Jazan 45142, Saudi Arabia; 5 Department of Pharmaceutics and Industrial Pharmacy, College of Pharmacy, Taif University, Taif 21974, Saudi Arabia; 6 Department of Clinical Laboratory Sciences, College of Applied Medical Sciences, King Khalid University, Abha 61421, Saudi Arabia; 7 Department of Pharmacy, Mohammed Al-Mana College for Medical Sciences, Safaa, Dammam 34222, Saudi Arabia; 8 Department of Pharmacognosy, College of Pharmacy, Prince Sattam Bin Abdulaziz University, P.O. Box 173, Al-Kharj 11942, Saudi Arabia; 9 Herbal Bioactive Research Laboratory, Faculty of Pharmacy, Integral University, Dasauli, Kursi Road, Lucknow 226026, Uttar Pradesh, India; 10 Department of Pharmacology, School of Medicine, Maldives National University, Male 20402, Maldives; 11 Department of Pharmaceutics, College of Pharmacy, King Khalid University, Abha 61421, Saudi Arabia

**Keywords:** probiotics, viral infections, respiratory viral infections, immunomodulatory effects, SARS-CoV-2, probiotics delivery

## Abstract

Mortality and morbidity from influenza and other respiratory viruses are significant causes of concern worldwide. Infections in the respiratory tract are often underappreciated because they tend to be mild and incapacitated. On the other hand, these infections are regarded as a common concern in clinical practice. Antibiotics are used to treat bacterial infections, albeit this is becoming more challenging since many of the more prevalent infection causes have acquired a wide range of antimicrobial resistance. Resistance to frontline treatment medications is constantly rising, necessitating the development of new antiviral agents. Probiotics are one of several medications explored to treat respiratory viral infection (RVI). As a result, certain probiotics effectively prevent gastrointestinal dysbiosis and decrease the likelihood of secondary infections. Various probiotic bacterias and their metabolites have shown immunomodulating and antiviral properties. Unfortunately, the mechanisms by which probiotics are effective in the fight against viral infections are sometimes unclear. This comprehensive review has addressed probiotic strains, dosage regimens, production procedures, delivery systems, and pre-clinical and clinical research. In particular, novel probiotics’ fight against RVIs is the impetus for this study. Finally, this review may explore the potential of probiotic bacterias and their metabolites to treat RVIs. It is expected that probiotic-based antiviral research would be benefitted from this review’s findings.

## 1. Introduction

Mortality and morbidity from influenza and other respiratory viruses are significant causes of concern worldwide [1,2]. A healthy immune system protects against viral infections and reduces susceptibility to subsequent bacterial infections [3,4]. Various variants of respiratory viruses are the cause of concern globally. As a result, new methods of regulating immune responses are required to defend against emerging respiratory viruses [5]. Probiotics are living microorganisms that, when provided in sufficient proportions, offer health advantages to their hosts, according to the World Health Organization (WHO) and the Food and Agriculture Organization (FAO) of the United Nations [6]. *Lactobacillus* and *Bifidobacterium* are prominent families of bacteria in the gut microbiota [7]. Probiotic formulations are microecological products that improve the intestinal flora’s architecture, diminish the growth of harmful microbes, and improve the immune response [8,9]. They modulate innate and adaptive immune responses, facilitating the immune system’s development and maturation. Probiotics regulate host-pathogen interactions by initiating the innate immune responses that comprise of Toll-like receptors (TLR), nuclear factor kappa B (NF-κB), mitogen-activated protein kinase (MAPK), and c-Jun NH2-terminal kinase (JNK) pathways. Probiotics such as *Lactobacillus* and *Bifidobacterium* can restore host health by eliminating pathogens and regulating immune responses in intestinal epithelial cells [6,10,11]. Probiotic strains are becoming popular due to their ability to modulate immunological responses, especially in the lower and upper respiratory tracts. Various studies have shown that probiotics regulate allergic reactions and protect the body against viral and bacterial infections [6,12,13,14,15,16,17].

In the present crisis of COVID-19, the immunomodulatory activities of probiotics may enhance the response to vaccines; therefore, probiotics could be a low-cost method of strengthening vaccination effectiveness and extending the protection period [18,19]. The gut–lung axis has been shown to have a role in improving gut health and homeostasis through the antiviral effects of oral probiotics. While various commercial probiotics have been shown to be beneficial against coronavirus, their efficacy in treating people infected with COVID-19 is still contested [20]. Probiotics have been shown to dramatically boost plasma cytokine levels, influenza vaccination effectiveness, and overall quality of life while decreasing virus titers and the frequency and duration of respiratory illnesses [21]. Probiotics and epithelial cells may directly influence cytokine responses and regulatory T cells [22]. Therefore, probiotics are promising candidates that should be studied for viral infections and immune function modulation. 

The gut immune system and treatment duration are linked to immunological disorders. Because probiotics’ benefits rely on the strains, clinical research findings have not been conclusive. Identifying specific target populations with greater susceptibilities to the possible impacts of probiotics may be necessary to test the effectiveness of these probiotics. The efficacy of active probiotic strains against RVIs is studied in this review. Moreover, probiotics as a treatment for respiratory infections, their mechanisms of action, clinical studies, probiotic delivery, and implications are also discussed. Conclusively, this study tries to investigate the therapeutic prospects of probiotic microorganisms and their metabolites as a treatment for RVIs. The results of this review are likely to help researchers in the field. 

## 2. Materials and Methods

The current comprehensive review compiled the information using diverse computerized databases such as Saudi Digital Library, ScienceDirect, Scopus, Google Scholar, and PubMed. Keywords such as *Bifidobacterium*, *Lactobacillus*, *Bifidobacterium* *longum*, *Bifidobacterium infantis*, *Bifidobacterium bifidum*, *Lactobacillus* species, lactic acid bacteria, *Lactobacillus del-brueckii*, *Lactobacillus fermentum*, *Lactobacillus reuteri*, *Lactobacillus johnsonii*, *Lactobacillus rhamnosus*, *Lactobacillus bulgaricus*, *Lactobacillus plantarum*, *Lactobacillus salivarius*, *Lactobacillus helveticus*, *Lactobacillus lactis*, *Lactobacillus casei*, *Lactobacillus acidophilus*, antiviral, health advantages, inflammatory bowel disease, allergies, inflammation, cytokines, allergic diseases, immunomodulatory, anti-inflammatory, anti-viral, innate or adaptive, innate immunity, anti-inflammatory cytokines, immunomodulatory effects, respiratory tract infections, respiratory viral infection, antiviral remedies, influenza, respiratory viruses, viral pneumonia, antiviral mechanism of action, SARS-CoV-2 infection, vaccine development, and clinical trials were used to search literature with respect to probiotics. Phrases such as “probiotic efficacy against viral infection”, “factors affecting the delivery of probiotics”, “dosage forms contained probiotic microorganism”, “effect of probiotics in the treatment of respiratory viral infections”, “antiviral effects of probiotics”, and “immunomodulatory effect of probiotics” were used to search the literature related to respiratory-related viral infection. 

Further information was retrieved from various medicinal books. For the comprehensive aspect, studies published in the last twenty-five years (from 1997 to 2022) were considered; however, there was no time limitation for the comprehensive review. Inclusion criteria were in vitro studies, in vivo studies, clinical studies, cross-sectional studies, cohort-type studies, and studies that addressed the treatment of respiratory viral infection with probiotics. Considering comorbidities, data related to respiratory viral infections and lung disease were included. Only studies available in English were included in this study; however, the selected studies should present reliable methodologies. Exclusion criteria were researched with dubious methods, master’s dissertations, unfinished research, and doctoral thesis.

## 3. Probiotic Bacteria Strains

**Figure 1:**
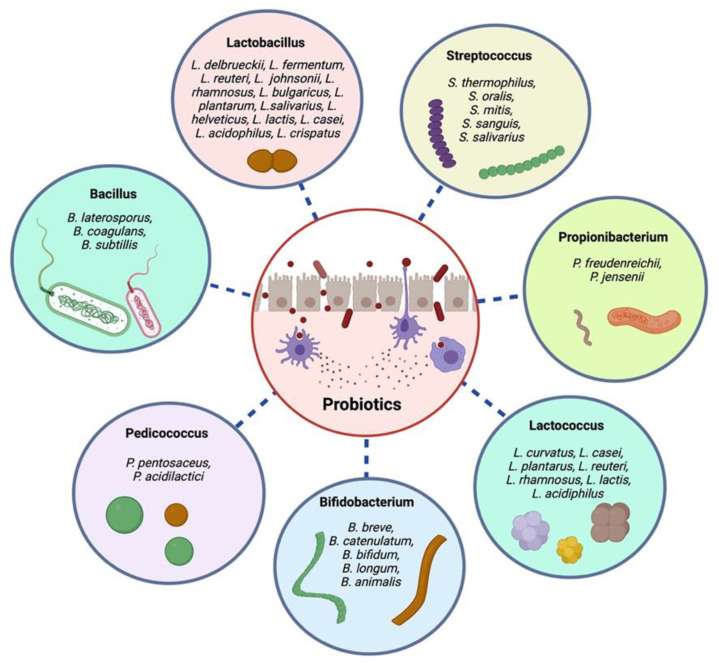
The most often utilized probiotic strains.

Probiotics are helpful living bacteria found in people and animals, whereas prebiotics is chemical substances that improve the growth of probiotics. Para probiotics and postbiotics refer to dead or inactivated living cells of probiotics and healthful metabolic products that are produced by the living cells of probiotics, respectively. However, probiotics and prebiotics have been scientifically shown to provide several physiological, functional, nutritional, and immunological advantages [23]. The most often utilized probiotic strains belong to *Bifidobacterium*, *Lactobacillus*, and *Streptococcus genera*. The *Bifidobacterium* strains include *Bifidobacterium longum*, *Bifidobacterium infantis*, and *Bifidobacterium bifidum*. *Lactobacillus* species include *Lactobacillus del-brueckii*, *Lactobacillus fermentum*, *Lactobacillus reuteri*, *Lactobacillus johnsonii*, *Lactobacillus rhamnosus*, *Lactobacillus bulgaricus*, *Lactobacillus plantarum*, *Lactobacillus salivarius*, *Lactobacillus helveticus*, *Lactobacillus lactis*, *Lactobacillus casei*, and *Lactobacillus acidophilus.* Other strains are *Enterococcus faecalis*, *Enterococcus faecium*, *Saccharomyces boulardii*, and *Streptococcus thermophilus* [24,25,26,27,28]. Commonly utilized probiotic strains are shown in Figure 1.

## 4. Probiotic Isolates and Their Health Advantages

Probiotics are fascinating more and more as alternatives to current and traditional medicines. Numerous anticipated mechanisms explore how probiotics work; they depend on probiotic strain, dosage, and intake route [29,30,31]. The adhesiveness ability of probiotics with the intestine mucosal layer is the most significant for immune system modulation and exhibits antagonistic results, and it has an antagonist effect contrary to pathogens [32,33,34]. Such precise adhesiveness belongs owing to the interaction between mucin and surface proteins; in that way, probiotics prevent pathogenic bacteria development and multiplication [35]. 

Furthermore, probiotics produce organic acids and bacteriocins that include lactic and acetic acids, leading to decreased pH intracellularly and raising the ionized organic acids, finally acting as killers of pathogens [36,37,38,39]. Probiotic bacteria produce biological activity by conquering pathogen-binding spots [40]. Bacteria interact with each other in their environment through chemical signaling molecules called autoinducers called quorum sensing. Signaling regulates the behavior of enteric microbes responsible for infections and colonization inside the host. *Lactobacillus* releases a molecule that constrains signaling of quorum sensing, directly interacts with signaling, or directly interacts with the *E. coli* O157 gene of bacterial transcription responsible for colonization [38,41,42,43,44]. Moreover, probiotic bacteria express their immunomodulatory action by interrelating with epithelial, dendritic cells, monocytes/macrophages, and lymphocytes [45]. These diverse mechanisms of action of probiotics make them the potential agent in preventing and treating numerous diseases such as cancer, diabetes, diarrhea, obesity, cardiac disorders, human immunodeficiency virus (HIV), inflammatory bowel disease (IBS), kidney diseases, allergies, etc. [30,46,47]. These potential health claims fascinate the researchers towards the novel development of probiotics. However, current modern medicine has several minor to significant side effects. The need of the hour is to search for an alternative with fewer side effects that is more therapeutic.

Allergies are some specific conditions produced via hypersensitivity of the immune system [48]. Probiotics treat allergies by curing the impaired digestive system by reducing inflammation, strengthening the gut lining, and stabilizing the immune system. In addition, probiotics alter the antigen’s structure and decrease their immunogenicity, pro-inflammatory cytokines generation, and intestinal permeability. Overall, probiotic actions play a significant role in preventing and treating allergic diseases [49,50,51]. For example, *Lactobacillus* GG and *L. rhamnosus* GG improve the signs of food allergies and substantially decrease the risk of allergic diseases [52]. Furthermore, probiotics are used in irritable bowel syndrome (IBS) through potent mechanisms that reduce epithelial binding and suppress the growth of pathogenic bacteria, antimicrobial constituents’ production, immunoregulation, and improved epithelial barrier function and immunoregulation. Thus, these are used to treat Crohn’s disease, ulcerative colitis, and pouchitis [53,54,55].

Cancer is a significant cause of ailment and death across the world. The natural adjuvant is the best key to treating this chronic disease [56,57,58]. Probiotic bacteria exhibit anticancer effects through a specific group of microbes, including *Bifidobacterium* and *Lactobacillus* species. They reduce the carcinogenic enzyme levels of the colonic through various defensive mechanisms, including antimutagenic organic acids production, improving the host immune system, intestinal permeability regulation, and microflora balance [59,60,61]. Moreover, evidence recommends that foodstuffs containing probiotic bacteria possibly contribute to cardiac disorder by reducing serum cholesterol levels and controlling blood pressure. Suggested mechanisms involved are cholesterol assimilation, interfering with cholesterol absorption in the gut. In addition, the fermentation process distresses the blood lipids and facilitates an antihypertensive result [62,63,64,65]. At the same time, probiotic bacteria have significant effects as anti-inflammatory and immunomodulatory behavior via boosting the dendritic cell’s pattern of maturation through discharging tumor necrosis factor-α (TNF-α) interleukin-12 (IL-12) and raising the IL-10 levels besides restraining the generation of pro-inflammatory cells. *Bifidobacteria* persist in the intestines and play a substantial role in health promotion. *Bifidobacteria* induces upregulation of IL-10 secretion by reducing CD40 and CD80 expression. Subsequently, immunomodulatory and anti-inflammatory activities were observed by enhancing the production of IL-10 [66,67].

Numerous studies discovered about hypoglycemic and anti-diabetic effects of *Lactobacillus* spp. [68]. Probiotics improve antioxidant enzyme actions such as superoxide dismutase, glutathione peroxidase, and catalase. Many strains of lactic acid bacteria (LAB) have revealed antioxidant activities through several mechanisms, including the chelation of metal ions, scavenging of reactive oxygen species (ROS), and enzyme inhibition [69,70]. In this way, probiotics exhibit defensive action through the antioxidant-linked potential measures to counter different diseases. However, probiotics’ properties are still a matter of consideration. Additional clinical studies are required to understand the exact mechanism of action of probiotics in different diseases. Different described probiotics available on the market are shown in Table 1.

## 5. Probiotics Effect on Viral Replication

Novel infectious illnesses and unexpected pathogenic potential may result from viral transmissions between animals and humans. These illnesses influence human health, the economy, and other aspects of a global society. Fever, dry cough, myalgia, dyspnea, and weariness are some of the symptoms that are associated with these disorders. Other symptoms include sore throat, rhinorrhea, headache, and gastrointestinal disorders. The most prevalent and severe sign of the illness seems to be pneumonia [71]. Unfortunately, there is no specific drug for the treatment of various new emerging viruses. In addition, the drug design and validation process, which is necessary for developing novel antiviral therapies, takes substantial time.

Consequently, repurposing natural compounds may provide alternatives and enhance antiviral treatment options. In addition, the particular function that probiotics play in regulating the microbes in the gut, maintaining gut homeostasis, and generating interferon as an antiviral mechanism is shown. The rapid mutation rates of viruses, particularly RNA viruses, make it challenging to develop effective treatments or vaccinations for viral illnesses. In this section, we evaluated the research on the antiviral effects of probiotics for preventing and treating viral infections based upon the different virus types.

### 5.1. Human Immunodeficiency Virus (HIV)

An in vitro investigation was conducted to assess the efficacy of LAB isolated from healthy women’s breastfeeding to prevent HIV-1 infection. There were 38 different types of breastmilk bacteria tested in this research investigation. Bacteria that have been heated to death and cell-free liquids from bacteria cultures were tested for their ability to stop HIV-1 infection. Viral isolates with tropism for CXCR4, CCR5, or dual tropism were used in the tests. These findings establish for the first time that commensal LAB isolated from human breastmilk suppresses HIV-1 infection in vitro and indicate that these bacteria may play a role in mucosal protection against HIV-1 in the nursing newborn [72].

### 5.2. Herpes Simplex Virus (HSV)

HSV-1 and HSV-2 were suppressed dose-dependently by *Enterococcus mundtii* ST4V, isolated from soya beans [73]. Enterocins ST4V and CRL35 inhibited viral particle replication [73,74]. Several vaginal *Lactobacillus strains* were tested in vitro for their ability to protect against the herpes simplex virus type 2 (HSV-2) infection. Bacterial cells that are still alive affect several stages of viral replication. Anti-HSV-2 activity lacked virucidal properties. It was exerted via the action of soluble bacterial factors, which could inhibit the generation of infective virions in the presence of the bacteria. Infected cells fed cell-free lactobacilli supernatants had considerably lower HSV-2 production. Lactic acid effectively inhibited viral intracellular antigen production, and both virucidal efficacy and replication inhibition were associated [75]. HSV-2 replication was inhibited in other investigations by a non-protein cell wall component of the bacteria *Lactobacillus brevis* [76]. Probiotic strains of *Lactobacillus paracasei* subsp. *rhamnosus*, *Lactobacillus paracasei*, *L. Plantarum*, and *Lactobacillus reuteri* entrapped vesicular stomatitis viruses by adhering to the particles [77].

### 5.3. Swine Influenza Virus

An investigation was conducted to determine the probiotic *Enterococcus faecium* (*E. faecium*) NCIMB 10415’s inhibitory impact on replicating two swine influenza virus strains (H1N1 and H3N2) in a continuous porcine macrophage cell line (3D4/21) and MDBK cells. The examinations showed direct adsorptive trapping of SwIV through *E. faecium*. A probiotic microorganism fights influenza viruses in at least two ways: directly interacting with them and boosting the body’s natural defenses at the cell level [78]. There are many ways that LAB probiotics can be antiviral: They can interact with viruses directly, make antiviral inhibitory metabolites, and make the immune system work more [79]. A high degree of specificity and selectivity was shown by LAB species for the inhibitory action [80]. IL-10 is an anti-inflammatory cytokine in the human immune response [80,81]. IL-10 was initially identified as a T-helper type 2 (Th2) cell product that suppressed cytokine generation in Th1 cells [82,83]. Additionally, probiotics may inhibit the production of pro-inflammatory cytokines by interfering with the mitogen-activated protein kinase (MAPK) and nuclear factor-kappa B (NF-κB) pathways [84,85].

Observations of virus replication inhibition have been the basis of most bacteria in antiviral activity reports. In addition, the particular function that probiotics play in regulating the microbes in the gut, maintaining gut homeostasis, and generating interferon as an antiviral mechanism is shown. Probiotics activate the macrophages and NK cells, modulate immunoreactions, and enhance the immune reaction to inhibit viruses [86,87]. A study has exhibited that either live LAB or cell-free supernatants (CFS) might restrict the porcine epidemic diarrhea virus (PEDV). The specific mechanism of probiotics is unclear and might be a powerful treatment against a pandemic strain of PEDV [88]. Another study has shown through the mice model that genetically engineered *Lactobacillus casei* oral vaccine effectively stimulates the mucosal SIgA and systemic IgG antibody responses [89]. Many types of research have been conducted to search for desired genes and antigens to fight the PEDV of probiotics [89,90,91,92]. In their examination, Liu et al. showed that modified *Lactobacillus plantarum* acted strongly as an antiviral agent in intestinal porcine epithelial cell lines [90]. The rapid mutation rates of viruses, particularly RNA viruses, make it challenging to develop effective treatments or vaccinations for viral illnesses. This is especially the case with RNA viruses. There is still much mystery surrounding the complex mechanism by which probiotics affect the host biology and immune system. Further studies are currently required to determine the precise mechanism of antiviral action.

## 6. Immunomodulatory Effects of Probiotic

Probiotics protect the host by regulating, stimulating, and modulating immune responses. To better understand probiotics’ immunomodulatory effects, researchers have focused on comprehending their potential better to prevent or relieve some illnesses for which effective medical therapy is currently lacking. It has been proven scientifically that immune cells (T cells and B cells) play a role in adaptive immunity. They protect the body from infections by building memories of the diseases they fight [93]. This section summarizes current research on probiotics’ immunomodulatory characteristics.

Probiotics have three functions such as protective, metabolic, and trophic [94]. It is worth mentioning that trophic function has gained attention in immunomodulation investigations. Generally, vertebrates’ immune systems may be classified as innate or adaptive. Innate immunity is a type of defense that is not specific. For example, when pathogens are found in the body, it reacts quickly or almost immediately. It also has a pathogen-specific long-term protective memory that helps the adaptive immune system fight and kills pathogens when they return [95]. Adaptive immune responses are triggered when lymphocytes, particularly B and T cells, recognize antigens with their unique receptors. There have been a lot of studies and reports about probiotics in the last few years. They have been found to help the immune system in many ways, including boosting the immune barrier [96,97]. Probiotic homogenates from *Lactobacillus acidophilus*, *Lactobacillus rhamnosus* GG, *Lactobacillus delbrueckii* subsp. *bulgaricus*, *Streptococcus thermophiles*, and *Bifidobacterium lactis* have been shown to inhibit mononuclear cell growth [98]. *Bifidobacterium breve* exhibits an elevated humoral immune response following stimulation with IgA, but *Bifidobacterium bifidum* dramatically improves antibody responses to ovalbumin [99].

Additionally, enterocytes and M cells may transport macromolecules, antigens, and microbes through the epithelium via a transepithelial vesicular transport mechanism. Antigenic chemicals boost the body’s innate and adaptive immune systems after getting through the intestinal barrier [100]. Strains of probiotic bacteria significantly impact the gut barrier by activating B cells to produce IgA, which helps maintain a healthy gut barrier. Probiotics have been shown to influence the generation of cytokines by APCs, which begins adaptive responses, an in vitro study using enterocyte cells (caco-2, HT-29, and dendritic cells derived from PBMC). Cytokines also help the body fight off bacteria, fungi, viruses, and other harmful things that try to get in. Based on research undertaken in animal models, it has been shown that specific nuclear-regulated cytokine genes benefit from cytokine-mediated binding and cascade activation by activating or inhibiting particular cell-surface receptors [101,102,103]. IL-1, IL-8, and TNF- were all increased by *Lactobacillus sakei* in an in vitro investigation involving Caco-2 cells; however, TGF- β (anti-inflammatory) production was affected by *Lactobacillus johnsonii*. This study found that interleukin-6 promotes the clonal proliferation of IgA B lymphocytes while stimulating the synthesis of antibodies such as immunoglobulin M and immunoglobulin G and decreasing IgE secretion [104]. Anti-inflammatory cytokines, such as IL-4, IL-5, IL-6, IL-10, and IL-13, are produced by Th2 cells. In addition, B cells, monocytes, DCs, and Tregs induce an adaptive immune response in the body [105,106]. A study was conducted to investigate the effect of *Lactobacillus bulgaricus*, *Lactobacillus casei*, and *Lactobacillus crispatus* on *Escherichia coli* to investigate bacterial modulating effects on cytokine responses. Probiotics interact with immune-competent cells and change the production of proinflammatory cytokines. This is because there was a significant drop in TNF-α output in inflamed mucosa grown with *L. casei* and *L. bulgaricus*. Interleukin-10 mouse models through the *Bifidobacterium infantis* and *Lactobacillus salivarius* were employed to assess their influence on host immune systems in the mucosa and systemic cytokine profiles [107].

Probiotic-treated animals had significantly lower interferon (IFN) and TNF (TNF-α) levels in their Peyer’s patch lymphocytes and spleen cells, respectively [108]. In the p38 MAPK pathway, *Lactobacillus rhamnosus* GG plays a critical function in activating anti-apoptotic Akt/protein kinase B and inhibiting pro-apoptotic factors [109]. *Bifidobacterium* and *Lactobacillus* species were given to rats to evaluate the immunomodulatory effects of probiotics, with overexpression of IL-10 (anti-inflammatory cytokines) and downregulation of TNF-α and IL-6 showing modulation or regulation of immune responses (proinflammatory cytokines). The studies showed that the probiotics significantly increase IgG and IgA concentrations in rats, although this is dose-dependent. Probiotics also can be immunomodulators to interact with epithelium and DCs, macrophages/monocytes, and lymphocytes [110]. Another study discovered that the proinflammatory cytokine TNF-α was significantly lowered by the *Lactobacillus bulgaricus* and *Lactobacillus casei* when interacting with immunocompetent cells. Numerous *Bifidobacterium* and *Lactobacillus* strains have been shown to induce TGF-β, IL-6, and IL-10 expression in epithelial cells and promote immunoglobulin synthesis further (IgA). These probiotic bacteria strains make the immunoglobulin receptors on the cells of the intestinal epithelium cells [111]. Studies show that probiotics can only affect cytokines from a single strain. Therefore, combining different probiotic strains to treat inflammation-related tissue damage and gastrointestinal inflammation in humans is beyond the scope of further research.

## 7. Role of Probiotics in Respiratory Tract Infections

Many non-antiviral and antiviral remedies are presently searched to conquer morbidity and mortality correlated with influenza and other respiratory virus infection [112,113]; still, no one is entirely effective in winning the fight against them. Modern medicine and nutraceutical have a broad spectrum of uses in various ailments, including antiviral, immunomodulators, antioxidant, hepatoprotective, anticancer, and cardioprotective [114]. Studies comparing the makeup of the lung microbiome in healthy and diseased states have shown substantial variations [115,116]. Lung disease has reduced bacterial diversity, with a single taxon or small group dominating [117,118]. It has been shown that age-related changes in composition and diversity in the gut microbiota were seen in cross-sectional research involving individuals from various age groups [119]. *Bifidobacteriaceae*, *Bacteroidaceae*, *Ruminococcaceae*, and *Lachnospiraceae* become less common with aging [119,120,121,122].

Unique microbial communities reside on the surfaces of mucosal linked with gastrointestinal tract (GT) and respiratory tract (RT), according to studies of the last 15 years, and these communities impact host defense against viral infections. The competent immune system lessens viral infections and the susceptivity to secondary bacterial infection. Antiviral immune responses induced by RVIs related to the change of microbial formation and activity (“dysbiosis”) in the GT and RT might change succeeding immune activity toward secondary bacterial infection or change the dynamics of inter-microbial communication; therefore, potential pathogenic bacterial species proliferation is increased. Thus, examining how respiratory viruses modify the gastrointestinal microbiome is worthwhile. It has been proposed, for example, that there is a link between viral-mediated inhibition of antibacterial immune responses [3]. The density of DNA and RNA viruses in the intestinal virome equals that of bacterial cells. It may result in a 20:1 enhancement in bacterial cells on gut mucosal surfaces and inside mucus layers [123]. Gut microbiome and respiratory infection interactions are bidirectional. Studies have shown that the respiratory virus may change the gut microbiome; it forms adaptive immune responses to fight the respiratory viruses. Antibiotic cocktail pretreatment in mice has raised morbidity from influenza infection [124,125]. Inhibiting severe illness and limiting viral load, the gut microbiota and immune system interact and perform a protective function. Immunity in elderly individuals and infants is weakened. As a result, these two categories have a high mortality rate and are easily infected by viruses.

**Figure 2:**
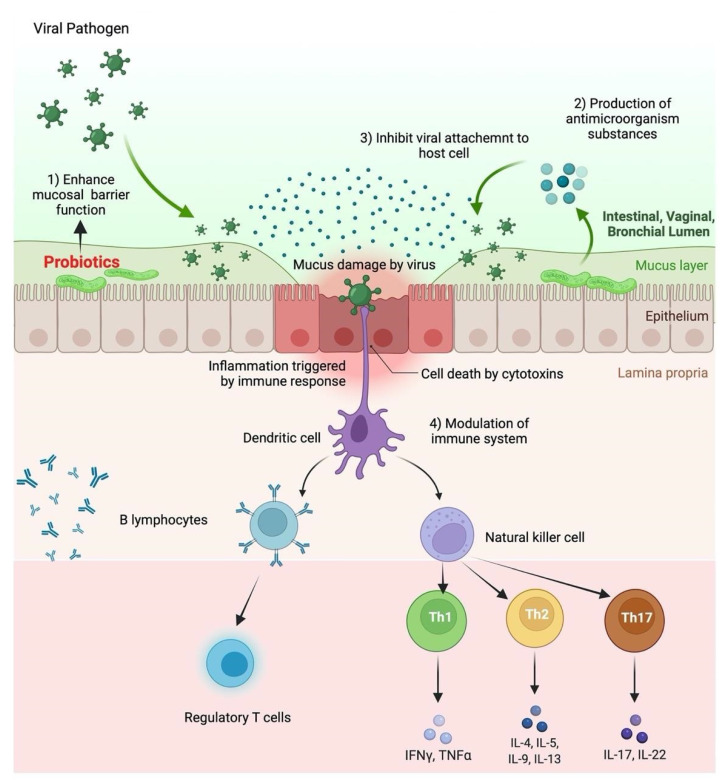
Probable antiviral mechanism of action of probiotics.

The usage of probiotics has extensively grown because of their effect on immune responses, especially for those affected by lower and upper respiratory tract infections. Cytokine storm is an inflammatory reaction of superinduction that has been associated directly with severe complications and viral pneumonia of respiratory diseases. Probiotics as potential immunomodulatory agents and enhance the host’s response to RVIs. Therefore, probiotics’ antiviral properties and immune responses are vital to understanding [108,109,126]. Furthermore, interactions between probiotics and macrophages and dendritic cells were seen in the lamina propria, resulting in NK cell activation, which triggers IFN-γ production to defend against viruses. PAMPs of probiotics interact with different TLRs, and NF-κB-mediated antiviral gene expression is incited. Efficient immune cells go to infection sites via circulatory and lymphatic systems to protect against respiratory viruses [5]. The probable antiviral action of probiotics is shown in Figure 2.

## 8. Role of Probiotics in SARS-CoV-2 Infection

In December 2019, the severe acute respiratory syndrome-corona virus-2 (SARS-CoV-2) was discovered in China’s Hubei province, and it causes COVID-19. SARS-CoV are enveloped, positive sense single-strand RNA viruses that belong to the broad family Coronaviridae and subfamily Caronavirinae [127]. COVID-19 has forced us to execute public health measures worldwide as no specific medications are in hand to treat this viral infection [128,129]. The risk of COVID-19 infection is higher than the seasonal flu [130]. COVID-19 is a viral infection that causes respiratory distress but can also induce signs and symptoms related to the gastrointestinal tract. The human gut microbiota regulates immune system homeostasis, which is vital for protecting responses to diverse infections. In addition, lung microbiota and gut have reciprocal interactions called the gut–lung axis (GLA). As a result, metabolites and gut microbiota may trigger any alterations undermining the immune system’s antiviral function against the respiratory virus, including SARA-CoV-2 [131]. Healthy lungs and gut microbiota protect against respiratory tract infection (RTI) related to COVID-19 and the influenza A virus. Thus, any changes in the gut microbiota can impair other organs’ activities [132,133]. Additionally, 33% of patients suffer from irritable syndrome (IBS), and 50% of patients with inflammatory bowel disease (IBD) are sensitive to respiratory ailments without chronic or acute respiratory complications [134,135].

The current pandemic needs alternative treatment to control the high morbidity and mortality rate. We can use previously approved remedies with safety profiles to treat this disease. Probiotics may be available as an alternative treatment with safety profiles. Some studies have been conducted against different strains of SARS-CoV-2, and results show that probiotics can manipulate cytokine storms and modulate immune responses [136,137]. It was found that various commercial probiotics are used and effective against SARS-CoV-2, but the subject of their efficacies is debatable in treating COVID-19 patients now. Nineteen clinical trials were found on ClinicalTrials.gov in the context of probiotics such as mixtures of *Lactobacillus*, *Bifidobacteria*, and *Lactobacillus* to treat COVID-19 patients [89]. The gut microbiota composition can change the prognosis and severity of COVID-19 during hospitalization and alter the immune responses [138]. A microbiome-oriented risk assessment might be used for severe patients’ risk profiles [139]. A few components of probiotics could effectively bind the ACE2 receptor proteins and spike proteins (S) to avert the virus from penetrating the host body [140]. It was also found that administering more than twenty probiotics has improved antiviral antibody production and anti-inflammatory interleukin levels and reduced the viral load [5,141,142,143].

**Figure 3:**
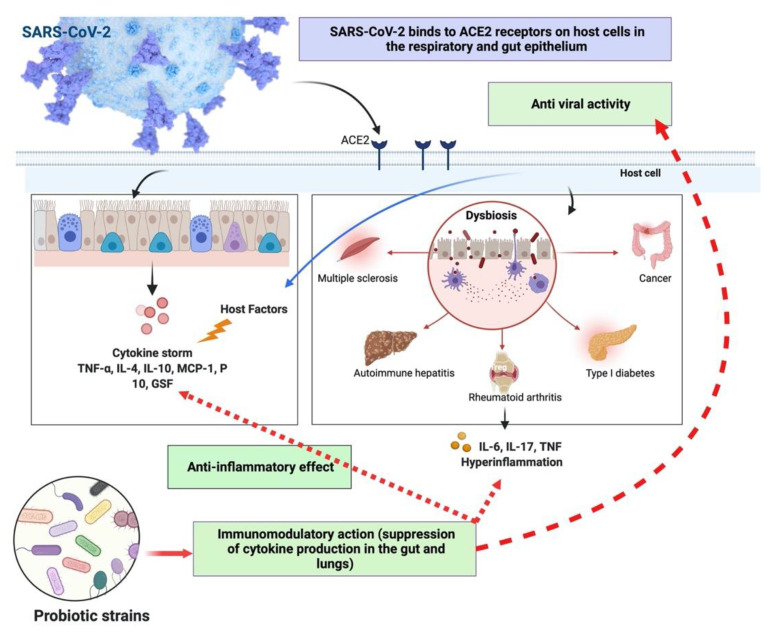
The possible role of probiotics in SARS-CoV-2 infection.

*Bifidobacterium* spp. and *Lactobacillus* spp. are vital traditional probiotics that can regulate a varied gut ecology in flight against SARS-CoV-2. The results of clinical trials and circumstantial evidence provide the foundation for the argument for using probiotics to treat SARS-CoV-2. In the gut microbiota, *Lactobacillus* may cause eubiosis via its antiviral properties, which can have an anti-inflammatory impact and help avoid superinfections. Probiotics have exhibited prospects for lessening the severity of symptoms and viral pathogenicity of COVID-19 and significant nourishing help for patients, but clinical approaches ought to be developed. Therefore, shortly can be enlightened on probiotics’ preventative or medicinal role. The possible role of probiotics in SARS-CoV-2 infection is shown in Figure 3.

## 9. Clinical Trial on Probiotics’ Role in Respiratory Viral Infections

Immunity and airway physiology could be modulated by intestinal microbiota through the gut–lung axis. The microbiome analysis of COVID-19 patients showed a specific intestinal dysbiosis in COVID-19 disease pathophysiology. COVID-19 might be controlled by managing the intestinal microbiome; therefore, the probiotic’s role is crucial in the present epidemic, but probiotics’ grounds for treating COVID-19 are indefinite. Researchers used an in vitro cytokine response test to analyze the immune system of probiotic lactic acid bacteria used to control COVID19 in a single-arm, double-blinded, prospective experiment. The emphasis of the study was to evaluate the effectiveness of *Bifidobacterium longum*, *Lactobacillus plantarum* (*L. plantarum*), and *Lactococcus lactis* ssp. *lactis*, against infection with respiratory RNA viruses. Twenty qualified volunteers were enrolled, and 18 of them finished the intervention. In vitro cytokine response assay showed a high innate cytokine index for all subjects by *L. plantarum*. From sixteen to eighteen subjects showed a rising level of cytokine index with significant differences in the fold change. At last, it was concluded in this trial that *L. plantarum* exhibited immunomodulatory effects and mimicked the blood cytokine responses developed by the initial immune response to viral infection. The trial outcomes confirmed that *L. plantarum* might be the potential alternative to manage COVID-19 [144].

In clinical trials on diabetes type 2 patients, it was found that fermented milk containing *L. acidophilus* (*L. acidophilus*) LA5 and *Bifidobacterium lactis* Bb12 control anti-inflammatory cytokines and help to improve blood sugar levels [145]. It also reported that probiotics reduce blood sugar levels and insulin resistance by improving inflammation. Yogurt with *L. acidophilus* strain La-5 and *Bifidobacterium animalis* (*B. animalis*) remarkably decreases HbA1c levels [68]. A clinical study showed that a lessening level of probiotics such as *Bifidobacterium* and *Lactobacillus* is the cause of the imbalance of intestine microbiota among some patients with COVID-19, which leads to secondary infection due to bacterial translocation [146]. Another study was conducted on 42 participants of two nursing homes with ages ≥ 65 years and administered jelly of 10 billion heat-killed *L. paracasei* or placebo for six weeks. Administration of the influenza vaccine is performed after three weeks of jelly intake. The outcomes have shown no significant variation between the groups in immune parameters with antibody responses against the vaccinations [147]. Studies have proven that intake of probiotics is good for lessening the RI symptoms and modulating the immune response. Therefore, RDBPC parallel-group trial was conducted to examine this activity. *L. casei* 431 and *L. paracasei* were received by 1104 healthy adults. After 21 days, participants were vaccinated with the seasonal influenza vaccine. The trial’s findings revealed that *L. casei* 431 had no meaningful influence on immunological responses to influenza vaccination but did alleviate UR symptoms [148].

According to the research, age-related immunological dysregulation increases infection rates and lowers vaccine efficiency. In a clinical trial, *L. casei* Shirota reduced respiratory symptoms in senior nursing home residents and boosted their immune response to influenza vaccination [149]. A clinical examination was conducted to examine whether a daily probiotic dairy drink may improve the immunological response to influenza vaccination in healthy senior volunteers over 70. Anti-influenza antibodies were more significant in the probiotic product group than in the control group. Individuals over 70 may benefit from frequent use of a specific probiotic product demonstrated to enhance particular antibody responses to influenza vaccination in this age range [150]. The studies enlisting the probiotic efficacy against viral infection are summarized in Table 2. According to our research evaluation, probiotics seem to be a cost-effective method of enhancing vaccination effectiveness and extending protection. Therefore, studies in the future should concentrate on finding the most promising strains, dosages, and timing of vaccination.

## 10. Scope of Prebiotics or Probiotics and Vaccine Development to Prevent Viral Infections

Prebiotics are a form of dietary fiber demonstrated to boost antibody levels after immunization. Epidemiologic studies have observed that the estimation of intake of prebiotics is complex, but quantifying dietary fiber is a routine process [156]. This section investigates the immunogenicity of prebiotics and probiotics. Prebiotics and probiotics are related in terms of their advantages. Many vegetables and fruits are a source of prebiotics, specifically those containing complex carbohydrates, such as resistant starch and fiber [157]. These complex carbohydrates are not digestible; therefore, they pass on by the digestive system as food for various microbes and bacteria. Prebiotics are similar to fertilizers in that they encourage the growth of beneficial bacteria in the digestive tract. Prebiotics has many beneficial effects that support a healthy gut for a better digestive system, lessen adverse antibiotic effects, and promote other benefits. However, it is found that less research has been conducted on prebiotics than on probiotics. Microbiome therapy describes the consumption of a mixture of prebiotics and probiotics. Prebiotic fibers assist feed and potentiate probiotic bacteria. The combination of these two enhance probiotics’ effectiveness.

Infectious illness prevention relies heavily on vaccines, which are unlikely to change soon [158]. Prebiotics alter the immune responses to the allergy and cause a lower incidence of dermatitis. Many studies have exhibited that prebiotic carbohydrate affects vaccine-specific antibody response to develop the immune system in healthy infants. A prebiotic mixture of long-chain fructo-oligosaccharides and short-chain galacto-oligosaccharides (scGOS) might work especially by stimulating or down-regulating Th1 and Th2 actions, respectively [159]. Current studies have indicated particular activities on normal T cells with upregulation and downregulation of Th1 and Th2, respectively [160,161,162]. Probiotics enrich adaptive and innate immunity [131,163].

Antibody responses to vaccines may be affected by probiotics due to their immunomodulatory activity. Therefore, the administration of probiotics in allergy-prone infants has increased. A systematic review was conducted to examine the effect of probiotics on RTI from January 2010 to January 2020. The results of this review concluded that probiotics could significantly raise cytokine levels in the plasma, improve the quality of life, and enhance the effect of the influenza vaccine by lessening the titer of viruses and duration and occurrence of RI. These immune-modulating and antiviral effects and their capability to provoke interferon production suggest using probiotics as an auxiliary cure to control COVID-19. Probiotics could be an appropriate therapy for RTI and a feasible option to help faster recovery with increased vaccine effect [21]. The immunomodulatory impacts of probiotics could affect the response to vaccines. Another systematic review analyzed the randomized placebo-controlled human research to examine probiotics’ consequences on humoral vaccine reactions. In this, 3812 enrolled people were examined through 26 studies to explore the outcome of 40 distinct probiotic strains on the reaction to 17 diverse vaccines. Probiotics have been demonstrated to have a beneficial effect in half of the investigations. The beneficial effect of vaccine response was most robust for parenteral influenza and oral vaccinations. There was a considerable variation between the selection of probiotics, dose, strain, purity, viability, and timing and duration of administration. The findings of this analysis indicated that probiotics are a very affordable intervention that may be used to increase vaccination efficacy and duration of protection [18].

**Figure 4:**
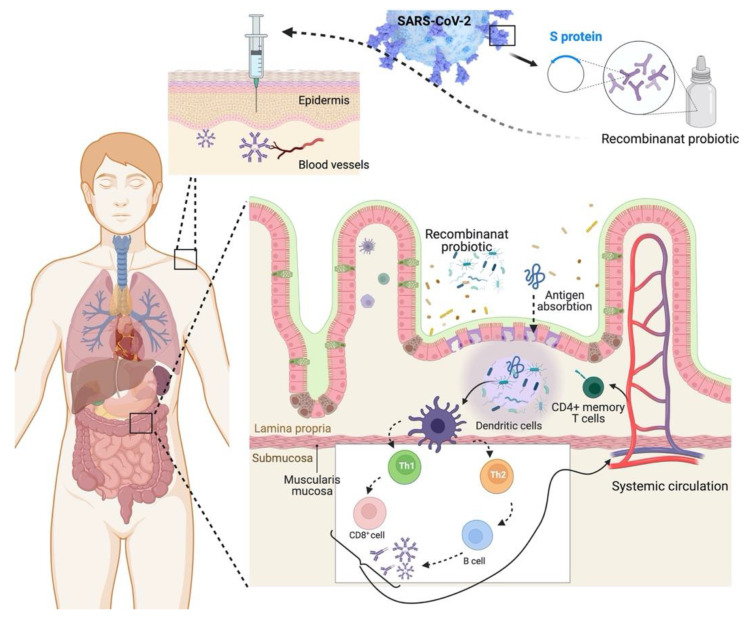
The possible scope of probiotics in vaccine development against viral infections.

Probiotics enriched the response to the influenza vaccine. Therefore, future researchers must search for the most favorable strains, timing, and administration doses concerning vaccination. *B. lactis* and *L. paracasei* (109 CFU) were administered for six weeks, and it increased the Influenza specific IgG, IgG1, and IgG3 levels (*p* ≤ 0.01). Higher seroconversion rates for influenza-specific IgG, IgG1, and IgG3 (*p* < 0.010) and higher influenza-specific IgA levels in saliva were noted 4 weeks following trivalent inactivated influenza vaccination (TIV) (*B. casei p* = 0.017, *B. lactis p* = 0.035) [154]. The live-attenuated influenza vaccine (LAIV) protects against influenza by activating the immune system’s mucosal mucosa. It is proven by the studies conducted on animals and adults that probiotics enhance the immune response when the vaccines are delivered mucosally [164]. From individual to individual, the immune response to vaccines differs. Therefore, the duration of the protection and efficacy against the various strains must examine. The above-discussed studies claimed that probiotics might enhance the vaccine efficacy and prolong the protection in the present scenario. Future research should confirm the optimal strains, timing, and administration doses of vaccination. The possible scope of probiotics in vaccine development against viral infections is shown in Figure 4.

## 11. Factors Affecting the Delivery of Probiotics

Probiotics efficiency depends on these microorganisms’ physiology, activity, and viability. In addition, not all bacteria are similar in terms of their advantages and ways of action. Therefore, not all probiotics are equivalent. Probiotic strains may have a wide range of beneficial benefits on the host, and most probiotic products have been developed using *Lactobacillus* and *Bifidobacterium* species to withstand the challenges of preparation, storage, and delivery [165]. Probiotic must be resilient enough to withstand the rigors of the manufacturing process without losing much of its vitality. The survival of microorganisms is affected by various physical and chemical conditions such as desiccation, oxygen exposure, humidity, osmotic pressure, and high temperature. In addition, various microbial species are affected by the severe GI conditions defined by low stomach pH and bile salts in the small intestine [166]. On the other hand, numerous *Lactobacillus* and *Bifidobacterium* species are microaerophilic and aero-tolerant and relatively tolerant of various environmental changes encountered during preparation, storage, and GI transportation, which, when combined with well-defined cultivation methods, makes them preferable probiotics products [167]. These microbes are hard to work with because they are prone to oxygen and often in gastric environments after indigestion [168,169]. Difficulties hinder next-generation probiotics in preserving these susceptible microorganisms’ viability via typical preparation, storage, and administration procedures. The capacity of a probiotic strain to outcompete another strain within a certain niche is thus required for the establishment of a probiotic in the gut to be long-lasting [170,171].

Oxygen-sensitive bacteria respond best to freeze-drying processing. It is necessary to desiccate oxygen-sensitive microorganisms to store them for an extended period [165]. Probiotic concentrations of 10^6^ CFU/mL in the small intestine and 10^8^ CFU/mL in the colon have been shown to have therapeutic benefits [172]. Several requirements must be satisfied to convey probiotic advantages to the consumer properly. From raw materials to the finished product must be monitored and documented for quality assurance, and this must be performed on time. The development of consumer products requires considerable expertise and experience. In the past, probiotic *Lactobacillus* and *Bifidobacteria* have been added to fermented dairy products, which have a short shelf-life and need refrigeration. Dietary supplements and “dry” food matrices may now include probiotics, which are projected to remain stable at room temperature and humidity for up to 24 months. High-quality probiotics may be effectively included in various delivery methods if the manufacturing process, product formulation, and strains are chosen correctly [173].

To produce the next generation of probiotics, new or improved methods of the microbial production cycle are required. Oral administration is the most effective when the intended site is in the GT. Other options include rectal and vaginal [24,59,170,172]. As a result of their high sensitivity to oxygen and the potential for gastrointestinal disorders after ingestion, working with these bacteria is complex. The difficulties in preserving the survival of these sensitive bacteria via typical preparation, storage, and distribution procedures pose a barrier to the commercialization of this sort of next-generation probiotics. A suppository base, such as silicon dioxide, microcrystalline cellulose, rice maltodextrin, magnesium stearate, methylcellulose, and hydroxypropyl, is added to improve the performance of the probiotic diluent, coating agent, lubricant, blinder, and sweetening agent. An oral probiotic composition should help prevent or cure respiratory infections. Understanding the function of the oral microbiota in infectious disease is critical for developing therapeutics for preventing and treating respiratory illnesses. However, further research is needed to determine the clinical effectiveness of delivery-related characteristics.

## 12. Conclusions

One of the most prevalent viral or bacterial infections is RVI. Because of an unbalanced microbial population in the digestive and respiratory systems, people are susceptible to RVIs. As far as the lungs are concerned, the gut microbiota plays a vital role in triggering immunological responses. Probiotic strains exhibit antiviral activity against common respiratory viruses. *Lactobacillus* and *Bifidobacteria* have been demonstrated to help in RVIs. Probiotic therapy may be advantageous in reducing disease-induced inflammation while also strengthening mucosal immunity and limiting the transmission of viral infections. Several research findings on probiotics suggest that their administration may also be beneficial in lowering the severity of RVIs and the significant difficulties associated with COVID-19. Probiotics can be used as a complementary therapy to reduce the mortality rate of COVID-19. The supplementation of probiotics may be beneficial in viral illnesses by enhancing immunity. An oral probiotic composition should help prevent or cure respiratory infections. Understanding the function of the oral microbiota in infectious disease is critical for developing therapeutics for preventing and treating respiratory illnesses. According to this review’s findings, probiotics’ immunomodulatory properties may help treat respiratory viral infections. Current microbial product processing procedures are not well adapted to produce next-generation probiotics; thus, improvements or new processing methodologies are required. Oral administration of next-generation live probiotics has received little research. However, further research is needed to determine the clinical effectiveness of delivery-related characteristics and the optimal dosage for each strain in various therapeutic settings. In our analysis of trials, probiotics seem to be a cost-effective method of enhancing vaccination effectiveness and extending protection. Future research should determine the most effective strains, dosages, and administration schedules concerning vaccinations. Finally, probiotic-based antiviral research is expected to benefit from this review’s findings.

## Figures and Tables

**Table 1:** Different described probiotics available on the market.

Brand	Product Benefits	Formulation	Strains
Boldfit	Immune support, digestive balance, weight loss, gut health	Each capsule has 30 billion CFU	*L. acidophilus* and *B. lactis*
Carbamide Forte Probiotics Supplement	Metabolism management	Each capsule has 30 billion CFU	*L. casei*, *L. plantarum*, *L. reuteri*, *L. salivarius*, *L. paracasei*, *B. bifidum*, *B. berve*, *B. lactis*, *S. boulardii*, *S. thermophilus*, and many more
HealthKart	Boost immunity by stimulating the activity of immune cells	Each capsule has 30 billion CFU	14 critical strains such as *L. plantarum*, *L. fermentum*, *L. reuteri*, *B. lactis*, *B. bifidum*, *B. boulardii*, *L. casei*, *L. acidophilus*, *S. thermophilus*, *B. berve*, *L. rhamnosus*, *B. lactis*, *L. paracasei*, and *L.salivarius*
Inlife	Digestive support and energy management	Each capsule has 2.75 billion CFU	*Lactobacillus acidophilus*, *Lactobacillus rhamnosus*, *Bifidobacterium bifidum*, *Bifidobacterium longum*, and *Saccharomyces boulardii*
Jarrow	Improve digestion, metabolism, absorption of nutrients, and immunity	Each capsule contains only about 5 billion CFU	*L. rhamnosus*, *L. helveticus*, *L. plantarum*, *L. lactis B. berve*, *Pediococcus acidilactici*, and *B. longum*
Mountainor	Enhances immunity and digestive health	Each capsule contains a total of 50 billion CFU with 16 carefully selected probiotic strains, which are most beneficial for gut health	It contains most strains from the L, B, and S category
Neuherbs Daily Probiotics	Stomach health support	Each capsule contains 20 billion CFUs	*Lactobacillus acidophilus*, *Lactobacillus fermentum*, *Lactobacillus rhamnosus*, *Bifidobacterium bifidum*, *Bifidobacterium longum*, and *Saccharomyces boulardii*
Now Foods	Digestive health support	Each capsule contains 25 billion CFU	The probiotic supplement contains all the necessary and imperative L, S, and B category acid strains
Swisse	Boosts immunity, healthy digestion, intestinal balance, growth of good bacteria, bloating, and flatulence	Each tablet contains 35 billion CFU	*B. lactis* and *L. acidophilus*
TrueBasics	Immune support	Each capsule contains 30 billion CFU	Lactic acids and *L. plantarum*
Wow	Immune support	Each capsule contains 20 billion CFU	*L. plantarum*, *L. casei*, *L. gasseri*, *B. bereve*, *B. infantis*, *L. fermentum*, *L. paracesi*, *L. acidophilus*, *B. bifidum*, *L. rhamnosus*, *L. salivarius*, *S. thermophilus*, *L. reuteri*, and *B. lactis*

**Table 2:** The studies enlisting the probiotic efficacy against viral infection.

Participants	Interventions	Comparison	Outcomes	Study Design	Reference
18	*L. plantarum*, *Bifidobacterium longum*, and *Lactococcus lactis* ssp.	*L. plantarum*, *Bifidobacterium longum*, and *Lactococcus lactis* ssp.	As an anti-COVID-19 probiotic, *L. plantarum* should be consumed daily	A single-arm, double-blind, prospective trial	[144]
20 infants	*Bifidobacteria* (*B. longum*/*B. infantis* and *B. breve*)	*Bifidobacteria* and placebo	Antipoliovirus reaction could be improved by intestinal *Bifidobacteria*	RPC trial	[151]
Infants (6 months of age)	Probiotic strains	Probiotics and placebo	Probiotics may boost the immune system’s response to Hib vaccination	RDPC, allergy-prevention trial	[152]
60	*Lactobacillus plantarum* CECT 7315/7316	*Lactobacillus plantarum* CECT 7315/7316 and placebo	It possesses immunostimulant properties and can improve influenza vaccine effectiveness in the elderly	RDPC, human trial	[153]
211	*Bifidobacterium animalis* ssp. *lactis* (BB-12^®^) & *Lactobacillus paracasei* ssp. *paracasei* (*L*. *casei* 431^®^)	BB-12^®^ (capsule) or *L. casei* 431^®^ and placebo	Immune function may be improved by using BB-12^®^ or *L. casei* 431^®^	RDPC, parallel-group study	[154]
42	*Lactobacillus paracasei*	*Lactobacillus paracasei* or placebo	Immune markers showed no significant changes	RDPC	[147]
1104 healthy adults	*L. paracasei* and *L. casei* 431	*L. paracasei* and *L. casei* 431 or placebo	*L. casei* 431 has no significant effect on influenza vaccination but lessens the period of URSs	RDPC, parallel-group study	[148]
15 adults	*Lactobacillus* in Jelly	*Lactobacillus* and placebo	*Lactobacillus* in Jelly improves the influenza vaccine effectiveness in the elderly	RPC trial	[155]
737 healthy people aged ≥ 65	*L. casei* Shirota	*L. casei* Shirota and placebo	It reduces respiratory symptoms and boosts the immune response to the influenza vaccine	RDBPC trial	[149]
308 elderly	*L. casei* DN-114 001	*L. casei* and placebo	Boost specific antibody responses to influenza vaccination	Two RDMC studies	[150]

Abbreviations: RDBPC—randomized, double-blind, placebo-controlled; RPC—randomized, placebo-controlled; UR—upper respiratory; IR—immune responses; URSs—upper respiratory symptoms; RMDC—randomized multicenter, double-blind, controlled; GI—gastrointestinal.

## References

[1] Mathew S., Smatti M.K., Al Ansari K., Nasrallah G.K., Al Thani A.A., Yassine H.M. (2019). Mixed Viral-Bacterial Infections and Their Effects on Gut Microbiota and Clinical Illnesses in Children. Sci. Rep..

[2] Wahab S., Annadurai S., Abullais S.S., Das G., Ahmad W., Ahmad M.F., Kandasamy G., Vasudevan R., Ali M.S., Amir M. (2021). Glycyrrhiza Glabra (Licorice): A Comprehensive Review on Its Phytochemistry, Biological Activities, Clinical Evidence and Toxicology. Plants.

[3] Hanada S., Pirzadeh M., Carver K.Y., Deng J.C. (2018). Respiratory Viral Infection-Induced Microbiome Alterations and Secondary Bacterial Pneumonia. Front. Immunol..

[4] Ahmad I., Wahab S., Nisar N., Dera A.A., Alshahrani M.Y., Abullias S.S., Irfan S., Alam M.M., Srivastava S. (2020). Evaluation of Antibacterial Properties of Matricaria Aurea on Clinical Isolates of Periodontitis Patients with Special Reference to Red Complex Bacteria. Saudi Pharm. J..

[5] Mahooti M., Miri S.M., Abdolalipour E., Ghaemi A. (2020). The Immunomodulatory Effects of Probiotics on Respiratory Viral Infections: A Hint for COVID-19 Treatment?. Microb. Pathog..

[6] Bermudez-Brito M., Plaza-Díaz J., Muñoz-Quezada S., Gómez-Llorente C., Gil A. (2012). Probiotic Mechanisms of Action. Ann. Nutr. Metab..

[7] Guarner F., Malagelada J.-R. (2003). Gut Flora in Health and Disease. Lancet.

[8] Zeng W., Shen J., Bo T., Peng L., Xu H., Ide Nasser M., Zhuang Q., Zhao M. (2019). Cutting Edge: Probiotics and Fecal Microbiota Transplantation in Immunomodulation. J. Immunol. Res..

[9] Hill C., Guarner F., Reid G., Gibson G.R., Merenstein D.J., Pot B., Morelli L., Canani R.B., Flint H.J., Salminen S. (2014). Expert Consensus Document: The International Scientific Association for Probiotics and Prebiotics Consensus Statement on the Scope and Appropriate Use of the Term Probiotic. Nat. Rev. Gastroenterol. Hepatol..

[10] Shang M., Chemistry J.S.-C. Medicinal; 2017, U. Vitamin D/VDR, Probiotics, and Gastrointestinal Diseases. Ingentaconnect.com.

[11] Plaza-Diaz J., Ruiz-Ojeda F.J., Gil-Campos M., Gil A. (2019). Mechanisms of Action of Probiotics. Adv. Nutr..

[12] Park M.-K., Ngo V., Kwon Y.-M., Lee Y.-T., Yoo S., Cho Y.-H., Hong S.-M., Hwang H.S., Ko E.-J., Jung Y.-J. (2013). Lactobacillus Plantarum DK119 as a Probiotic Confers Protection against Influenza Virus by Modulating Innate Immunity. PLoS ONE.

[13] Yasui H., Kiyoshima J., Hori T. (2004). Reduction of Influenza Virus Titer and Protection against Influenza Virus Infection in Infant Mice Fed Lactobacillus Casei Shirota. Am. Soc. Microbiol..

[14] Ohno H., Tsunemine S., Isa Y., Shimakawa M., Yamamura H. (2005). Oral Administration of Bifidobacterium Bifidum G9-1 Suppresses Total and Antigen Specific Immunoglobulin E Production in Mice. Biol. Pharm. Bull..

[15] Cross M., Immunology H.G.-I. (2001). Can Immunoregulatory Lactic Acid Bacteria Be Used as Dietary Supplements to Limit Allergies?. Int. Arch. Allergy Immunol..

[16] Isolauri E., Kirjavainen P.V., Salminen S. (2002). Probiotics: A Role in the Treatment of Intestinal Infection and Inflammation?. Gut.

[17] Hajavi J., Esmaeili S.-A., Varasteh A.-R., Vazini H., Atabati H., Mardani F., Momtazi-Borojeni A.A., Hashemi M., Sankian M., Sahebkar A. (2018). The Immunomodulatory Role of Probiotics in Allergy Therapy. Wiley Online Libr..

[18] Zimmermann P., Curtis N. (2018). The Influence of Probiotics on Vaccine Responses—A Systematic Review. Vaccine.

[19] Ahmad M.F., Ali M.S., Alsayegh A.R.A., Ahmad S., Alam N., Wahab S., Ali M.S., Athar M.T. (2021). A Current Novel Perspective Approach for Coronavirus Disease-2019 Pandemic Outbreak. J. Adv. Pharm. Technol. Res..

[20] Hung Y.P., Lee C.C., Lee J.C., Tsai P.J., Ko W.C. (2021). Gut Dysbiosis during COVID-19 and Potential Effect of Probiotics. Microorganisms.

[21] Darbandi A., Asadi A., Ghanavati R., Afifirad R., Darb Emamie A., Kakanj M., Talebi M. (2021). The Effect of Probiotics on Respiratory Tract Infection with Special Emphasis on COVID-19: Systemic Review 2010–20. Int. J. Infect. Dis..

[22] Marteau P., Shanahan F. (2003). Basic Aspects and Pharmacology of Probiotics: An Overview of Pharmacokinetics, Mechanisms of Action and Side-Effects. Best Pract. Res. Clin. Gastroenterol..

[23] Khaled J.M.A. (2021). Probiotics, Prebiotics, and COVID-19 Infection: A Review Article. Saudi J. Biol. Sci..

[24] Mishra S., Rath S., Mohanty N. (2020). Probiotics—A Complete Oral Healthcare Package. J. Integr. Med..

[25] Samot J., Badet C. (2013). Antibacterial Activity of Probiotic Candidates for Oral Health. Anaerobe.

[26] Scannapieco F.A. (2013). The Oral Microbiome: Its Role in Health and in Oral and Systemic Infections. Clin. Microbiol. Newsl..

[27] Russell D.A., Ross R.P., Fitzgerald G.F., Stanton C. (2011). Metabolic Activities and Probiotic Potential of Bifidobacteria. Int. J. Food Microbiol..

[28] Khalid M., Alqarni M.H., Wahab S., Annadurai S., Alamri M.A., Foudah A.I., Aljarba T.M., Akhtar J., Badruddeen, Ahmad S. (2022). Ameliorative Sexual Behavior and Phosphodiesterase-5 Inhibitory Effects of Spondias Mangifera Fruit Extract in Rodents: In Silico, In Vitro, and In Vivo Study. J. Clin. Med..

[29] Wang M., Liu P., Kong L., Xu N., Lei H. (2021). Promotive Effects of Sesamin on Proliferation and Adhesion of Intestinal Probiotics and Its Mechanism of Action. Food Chem. Toxicol..

[30] Hamasalim H.J. (2015). The Impact of Some Widely Probiotic (Iraqi Probiotic) on Health and Performance. J. Biosci. Med..

[31] Ahmad M.F., Wahab S., Ahmad F.A., Ashraf S.A., Abullais S.S., Saad H.H. (2022). Ganoderma Lucidum: A Potential Pleiotropic Approach of Ganoderic Acids in Health Reinforcement and Factors Influencing Their Production. Fungal. Biol. Rev..

[32] Hirano J., Yoshida T., Sugiyama T., Koide N., Mori I., Yokochi T. (2003). The Effect of Lactobacillus Rhamnosus on Enterohemorrhagic Escherichia Coli Infection of Human Intestinal Cells in Vitro. Microbiol. Immunol..

[33] Schiffrin E.J., Brassart D., Servin A.L., Rochat F., Donnet-Hughes A. (1997). Immune Modulation of Blood Leukocytes in Humans by Lactic Acid Bacteria: Criteria for Strain Selection. Am. J. Clin. Nutr..

[34] Amir M., Zafar A., Ahmad R., Ahmad W., Sarafroz M., Khalid M., Ghoneim M.M., Alshehri S., Wahab S., Ahmad S. (2022). Quality Control Standardization, Contaminant Detection and In Vitro Antioxidant Activity of Prunus Domestica Linn. Fruit. Plants.

[35] Kannan S., Balakrishnan J., Govindasamy A. (2020). Listeria Monocytogens—Amended Understanding of Its Pathogenesis with a Complete Picture of Its Membrane Vesicles, Quorum Sensing, Biofilm and Invasion. Microb. Pathog..

[36] Chang H.M., Foo H.L., Loh T.C., Lim E.T.C., Abdul Mutalib N.E. (2021). Comparative Studies of Inhibitory and Antioxidant Activities, and Organic Acids Compositions of Postbiotics Produced by Probiotic Lactiplantibacillus Plantarum Strains Isolated From Malaysian Foods. Front. Vet. Sci..

[37] Alakomi H.L., Skyttä E., Saarela M., Mattila-Sandholm T., Latva-Kala K., Helander I.M. (2000). Lactic Acid Permeabilizes Gram-Negative Bacteria by Disrupting the Outer Membrane. Appl. Environ. Microbiol..

[38] Wahab S., Muzammil K., Nasir N., Khan M.S., Ahmad M.F., Khalid M., Ahmad W., Dawria A., Reddy L.K.V., Busayli A.M. (2022). Review Advancement and New Trends in Analysis of Pesticide Residues in Food: A Comprehensive Review. Plants.

[39] Ahmad W., Yusuf M., Ahmad A., Hassan Y.A., Amir M., Wahab S. (2022). Development and Validation of Ultra Performance Liquid Chromatography (UPLC) Method for the Quantitative Estimation of Caffeine in Non-Alcoholic Soft and Energy Drinks. J. AOAC Int..

[40] Wu X., Vallance B.A., Boyer L., Bergstrom K.S.B., Walker J., Madsen K., O’Kusky J.R., Buchan A.M., Jacobson K. (2008). Saccharomyces Boulardii Ameliorates Citrobacter Rodentium -Induced Colitis through Actions on Bacterial Virulence Factors. Am. J. Physiol. Liver Physiol..

[41] Saeki E.K., Kobayashi R.K.T., Nakazato G. (2020). Quorum Sensing System: Target to Control the Spread of Bacterial Infections. Microb. Pathog..

[42] To H.T.A., Chhetri V., Settachaimongkon S., Prakitchaiwattana C. (2022). Stress Tolerance-Bacillus with a Wide Spectrum Bacteriocin as an Alternative Approach for Food Bio-Protective Culture Production. Food Control..

[43] Medellin-Peña M.J., Wang H., Johnson R., Anand S., Griffiths M.W. (2007). Probiotics Affect Virulence-Related Gene Expression in Escherichia Coli O157:H7. Appl. Environ. Microbiol..

[44] Prakash O., Usmani S., Gupta A., Jafri A., Ullah M.F., Wahab S., Arshad M., Kumar S. (2021). Bioactive Extracts of Ziziphus Mauritiana Induces Apoptosis in A549 Human Lung Epithelial Carcinoma Cells through the Generation of Reactive Oxygen Species. Curr. Cancer Ther. Rev..

[45] Gómez-Llorente C., Muñoz S., Gil A. (2010). Role of Toll-like Receptors in the Development of Immunotolerance Mediated by Probiotics. Proc. Nutr. Soc..

[46] Bajaj B.K., Claes I.J.J., Lebeer S. (2015). Functional Mechanisms of Probiotics. J. Microbiol. Biotechnol. Food Sci..

[47] Rajagopalan P., Wahab S., Dera A., Chandramoorthy H., Irfan S., Patel A., Abullias S., Zaman G., Ahmad I. (2020). Anti-Cancer Activity of Ethanolic Leaf Extract of Salvia Officinalis against Oral Squamous Carcinoma Cells in Vitro via Caspase Mediated Mitochondrial Apoptosis. Pharmacogn. Mag..

[48] Waserman S., Shah A., Cruikshank H., Avilla E. (2022). Recognition and Management of Food Allergy and Anaphylaxis in the School and Community Setting. Immunol. Allergy Clin. North Am..

[49] Gowri R.S., Meenambigai P., Prabhavathi P., Raja Rajeswari P., Yesudoss L.A. (2016). Probiotics and Its Effects on Human Health-A Review. Int. J. Curr. Microbiol. Appl. Sci..

[50] Abatenh E., Gizaw B., Tsegay Z., Tefera G., Aynalem E. (2018). Health Benefits of Probiotics. J. Food Sci. Technol..

[51] Ahmad I., Alshahrani M.Y., Wahab S., Al-Harbi A.I., Nisar N., Alraey Y., Alqahtani A., Mir M.A., Irfan S., Saeed M. (2022). Zinc Oxide Nanoparticle: An Effective Antibacterial Agent against Pathogenic Bacterial Isolates. J. King Saud. Univ. Sci..

[52] Licciardi P.V., Ismail I.H., Balloch A., Mui M., Hoe E., Lamb K., Tang M.L.K. (2013). Maternal Supplementation with LGG Reduces Vaccine-Specific Immune Responses in Infants at High-Risk of Developing Allergic Disease. Front. Immunol..

[53] Mikov M., Stojancevic M., Bojic G. (2014). Probiotics as a Promising Treatment for Inflammatory Bowel Disease. Hosp. Pharmacol. Int. Multidiscip. J..

[54] Toumi R., Samer A., Soufli I., Rafa H., Touil-Boukoffa C. (2021). Role of Probiotics and Their Metabolites in Inflammatory Bowel Diseases (IBDs). Gastroenterol. Insights.

[55] Jadhav V., Bhagare A., Wahab S., Lokhande D., Vaidya C., Dhayagude A., Khalid M., Aher J., Mezni A., Dutta M. (2022). Green Synthesized Calcium Oxide Nanoparticles (CaO NPs) Using Leaves Aqueous Extract of Moringa Oleifera and Evaluation of Their Antibacterial Activities. J. Nanomater..

[56] Ahmad M.F. (2020). Ganoderma Lucidum: A Rational Pharmacological Approach to Surmount Cancer. J. Ethnopharmacol..

[57] Wahab S., Alshahrani M.Y., Ahmad M.F., Abbas H. (2021). Current Trends and Future Perspectives of Nanomedicine for the Management of Colon Cancer. Eur. J. Pharmacol..

[58] Wahab S., Hussain A., Farooqui A.H.A., Parwez Ahmad M. (2013). Authentication and Quality Evaluation of an Important Ayurvedic Drug Averrhoa Carambola Linn Leaves. Asian J. Pharm. Clin. Res..

[59] Kumar M., Kumar A., Nagpal R., Mohania D., Behare P., Verma V., Kumar P., Poddar D., Aggarwal P.K., Henry C.J.K. (2010). Cancer-Preventing Attributes of Probiotics: An Update. Int. J. Food Sci. Nutr..

[60] Jampílek J., Kráľová K., Bella V. (2022). Probiotics and Prebiotics in the Prevention and Management of Human Cancers (Colon Cancer, Stomach Cancer, Breast Cancer, and Cervix Cancer). Probiotics in the Prevention and Management of Human Diseases.

[61] Ahmad M.F., Ahmad F.A., Ashraf S.A., Saad H.H., Wahab S., Khan M.I., Ali M., Mohan S., Hakeem K.R., Athar M.T. (2021). An Updated Knowledge of Black Seed (*Nigella Sativa Linn.*): Review of Phytochemical Constituents and Pharmacological Properties. J. Herb. Med..

[62] Sanders M.E., Klaenhammer T.R. (2001). Invited Review. The Scientific Basis of Lactobacillus Acidophilus NCFM Functionality as a Probiotic. J. Dairy Sci..

[63] Kechagia M., Basoulis D., Konstantopoulou S., Dimitriadi D., Gyftopoulou K., Skarmoutsou N., Fakiri E.M. (2013). Health Benefits of Probiotics: A Review. ISRN Nutr..

[64] Ramos C.L., Esteves E.A., Prates R.P., Moreno L.G., Santos C.S. (2022). Probiotics in the Prevention and Management of Cardiovascular Diseases with Focus on Dyslipidemia. Probiotics in the Prevention and Management of Human Diseases.

[65] Wahab S., Ahmad I., Irfan S., Ahmad M.F., Usmani S., Shoaib A., Ahmad W. (2021). Hydrogel: An Encouraging Nanocarrier System for the Delivery of Herbal Bioactive Compounds. Curr. Nanosci..

[66] Madsen K. (2006). Probiotics and the Immune Response. J. Clin. Gastroenterol..

[67] Abdolalipour E., Mahooti M., Salehzadeh A., Torabi A., Mohebbi S.R., Gorji A., Ghaemi A. (2020). Evaluation of the Antitumor Immune Responses of Probiotic Bifidobacterium Bifidum in Human Papillomavirus-Induced Tumor Model. Microb. Pathog..

[68] Andrade-Velásquez A., Domínguez-Cañedo L., Melgar-Lalanne G. (2021). Growth Kinetic Model, Antioxidant and Hypoglycemic Effects at Different Temperatures of Potential Probiotic Lactobacillus Spp.. Rev. Mex. Ing. Quim..

[69] Parle M., Malik J. (2014). Curd: A Sedative with a Bonus Bowl of Useful Side Effects. Int. Res. J. Pharm..

[70] Shori A.B., Aljohani G.S., Al-zahrani A.J., Al-sulbi O.S., Baba A.S. (2022). Viability of Probiotics and Antioxidant Activity of Cashew Milk-Based Yogurt Fermented with Selected Strains of Probiotic Lactobacillus Spp.. LWT.

[71] Sundararaman A., Ray M., Ravindra P.V., Halami P.M. (2020). Role of Probiotics to Combat Viral Infections with Emphasis on COVID-19. Appl. Microbiol. Biotechnol..

[72] Martín V., Maldonado A., Fernández L., Rodríguez J.M., Connor R.I. (2010). Inhibition of Human Immunodeficiency Virus Type 1 by Lactic Acid Bacteria from Human Breastmilk. Breastfeed. Med..

[73] Todorov S.D., Wachsman M.B., Knoetze H., Meincken M., Dicks L.M.T. (2005). An Antibacterial and Antiviral Peptide Produced by Enterococcus Mundtii ST4V Isolated from Soya Beans. Int. J. Antimicrob. Agents.

[74] Wachsman M.B., Farías M.E., Takeda E., Sesma F., de Ruiz Holgado A.P., de Torres R.A., Coto C.E. (1999). Antiviral Activity of Enterocin CRL35 against Herpesviruses. Int. J. Antimicrob. Agents.

[75] Conti C., Malacrino C., Mastromarino P. (2009). Inhibition of Herpes Simplex Virus Type 2 by Vaginal Lactobacilli. J. Physiol. Pharmacol..

[76] Mastromarino P., Cacciotti F., Masci A., Mosca L. (2011). Antiviral Activity of Lactobacillus Brevis towards Herpes Simplex Virus Type 2: Role of Cell Wall Associated Components. Anaerobe.

[77] Botić T., Klingberg T.D., Weingartl H., Cencič A. (2007). A Novel Eukaryotic Cell Culture Model to Study Antiviral Activity of Potential Probiotic Bacteria. Int. J. Food Microbiol..

[78] Wang Z., Chai W., Burwinkel M., Twardziok S., Wrede P., Palissa C., Esch B., Schmidt M.F.G. (2013). Inhibitory Influence of Enterococcus Faecium on the Propagation of Swine Influenza A Virus in Vitro. PLoS ONE.

[79] Al Kassaa I., Hober D., Hamze M., Chihib N.E., Drider D. (2014). Antiviral Potential of Lactic Acid Bacteria and Their Bacteriocins. Probiotics Antimicrob. Proteins.

[80] Serkedjieva J., Danova S., Ivanova I. (2000). Antiinfluenza Virus Activity of a Bacteriocin Produced by *Lactobacillus delbrueckii*. Appl. Biochem. Biotechnol..

[81] Alshahrani M.Y., Alfaifi M., Al Shahrani M., Alshahrani A.S., Alkhathami A.G., Dera A.A., Ahmad I., Wahab S., Beg M.M.A., Hakamy A. (2021). Increased MRNA Expression of Key Cytokines among Suspected Cases of Pneumocystis Jirovecii Infection. BMC Infect. Dis..

[82] Lalani I., Bhol K., Ahmed A.R. (1997). Interleukin-10: Biology, Role in Inflammation and Autoimmunity. Ann. Allergy, Asthma Immunol..

[83] Alsayari A., Muhsinah A.B., Almaghaslah D., Annadurai S., Wahab S. (2021). Pharmacological Efficacy of Ginseng against Respiratory Tract Infections. Molecules.

[84] Yoon S., Practice J.S.-G. (2011). Probiotics, Nuclear Receptor Signaling, and Anti-Inflammatory Pathways. Gastroenterol. Res. Pract..

[85] Wahab S., Ahmad I., Irfan S., Siddiqua A., Usmani S., Ahmad M.P. (2021). Pharmacological Efficacy and Safety of Glycyrrhiza Glabra in the Treatment of Respiratory Tract Infections. Mini Reviews Med. Chem..

[86] Maragkoudakis P.A., Chingwaru W., Gradisnik L., Tsakalidou E., Cencic A. (2010). Lactic Acid Bacteria Efficiently Protect Human and Animal Intestinal Epithelial and Immune Cells from Enteric Virus Infection. Int. J. Food Microbiol..

[87] Cha M.K., Lee D.K., An H.M., Lee S.W., Shin S.H., Kwon J.H., Kim K.J., Ha N.J. (2012). Antiviral Activity of Bifidobacterium Adolescentis SPM1005-A on Human Papillomavirus Type 16. BMC Med..

[88] Sirichokchatchawan W., Temeeyasen G., Nilubol D., Prapasarakul N. (2018). Protective Effects of Cell-Free Supernatant and Live Lactic Acid Bacteria Isolated from Thai Pigs Against a Pandemic Strain of Porcine Epidemic Diarrhea Virus. Probiotics Antimicrob. Proteins.

[89] Wang X., Wang L., Huang X., Ma S., Yu M., Shi W., Qiao X., Tang L., Xu Y., Li Y. (2017). Oral Delivery of Probiotics Expressing Dendritic Cell-Targeting Peptide Fused with Porcine Epidemic Diarrhea Virus COE Antigen: A Promising Vaccine Strategy against PEDV. Viruses.

[90] Liu Y.S., Liu Q., Jiang Y.L., Yang W.T., Huang H.B., Shi C.W., Yang G.L., Wang C.F. (2020). Surface-Displayed Porcine IFN-Λ3 in Lactobacillus Plantarum Inhibits Porcine Enteric Coronavirus Infection of Porcine Intestinal Epithelial Cells. J. Microbiol. Biotechnol..

[91] Wang X.N., Wang L., Zheng D.Z., Chen S., Shi W., Qiao X.Y., Jiang Y.P., Tang L.J., Xu Y.G., Li Y.J. (2018). Oral Immunization with a Lactobacillus Casei-Based Anti-Porcine Epidemic Diarrhoea Virus (PEDV) Vaccine Expressing Microfold Cell-Targeting Peptide Co1 Fused with the COE Antigen of PEDV. J. Appl. Microbiol..

[92] Ma S., Wang L., Huang X., Wang X., Chen S., Shi W., Qiao X., Jiang Y., Tang L., Xu Y. (2018). Oral Recombinant Lactobacillus Vaccine Targeting the Intestinal Microfold Cells and Dendritic Cells for Delivering the Core Neutralizing Epitope of Porcine Epidemic Diarrhea Virus. Microb. Cell Fact..

[93] Azad M.A.K., Sarker M., Wan D. (2018). Immunomodulatory Effects of Probiotics on Cytokine Profiles. Biomed Res. Int..

[94] Küskü-Kiraz Z., Genc S., Bekpınar S., Ünlücerci Y., Çevik A., Olgaç V., Gürdöl F., Uysal M. (2018). Effects of Betaine Supplementation on Nitric Oxide Metabolism, Atherosclerotic Parameters, and Fatty Liver in Guinea Pigs Fed a High Cholesterol plus Methionine Diet. Nutrition.

[95] Tan C., Wei H., Sun H., Ao J., Long G., Jiang S., Peng J. (2015). Effects of Dietary Supplementation of Oregano Essential Oil to Sows on Oxidative Stress Status, Lactation Feed Intake of Sows, and Piglet Performance. Biomed Res. Int..

[96] Wood C., Keeling S., Bradley S., Johnson-Green P., Green-Johnson J.M. (2007). Interactions in the Mucosal Microenvironment: Vasoactive Intestinal Peptide Modulates the down-Regulatory Action of Lactobacillus Rhamnosus on LPS-Induced Interleukin-8 Production by Intestinal Epithelial Cells. Microb. Ecol. Health Dis..

[97] Gill H.S., Cross M.L., Rutherfurd K.J., Gopal P.K. (2001). Dietary Probiotic Supplementation to Enhance Cellular Immunity in the Elderly. Br. J. Biomed. Sci..

[98] Kankaanpää P., Sütas Y., Salminen S., Isolauri E. (2003). Homogenates Derived from Probiotic Bacteria Provide Down-Regulatory Signals for Peripheral Blood Mononuclear Cells. Food Chem..

[99] Bodera P., Chcialowski A. (2009). Immunomodulatory Effect of Probiotic Bacteria. Recent Pat. Inflamm. Allergy Drug Discov..

[100] Snoeck V., Goddeeris B., Cox E. (2005). The Role of Enterocytes in the Intestinal Barrier Function and Antigen Uptake. Microbes Infect..

[101] Biswas G., Korenaga H., Nagamine R., Kawahara S., Takeda S., Kikuchi Y., Dashnyam B., Yoshida T., Kono T., Sakai M. (2013). Elevated Cytokine Responses to Vibrio Harveyi Infection in the Japanese Pufferfish (Takifugu Rubripes) Treated with Lactobacillus Paracasei Spp. Paracasei (06TCa22) Isolated from the Mongolian Dairy Product. Fish Shellfish Immunol..

[102] Mulder I.E., Wadsworth S., Secombes C.J. (2007). Cytokine Expression in the Intestine of Rainbow Trout (Oncorhynchus Mykiss) during Infection with Aeromonas Salmonicida. Fish Shellfish Immunol..

[103] Wahab S., Hussain A. (2013). Cytokines as Targets for Immunomodulation. Int. J. Pharm. Pharm. Sci..

[104] Galdeano C.M., de Moreno de LeBlanc A., Vinderola G., Bonet M.E.B., Perdigón G. (2007). Proposed Model: Mechanisms of Immunomodulation Induced by Probiotic Bacteria. Clin. Vaccine Immunol..

[105] Moore K.W., de Waal Malefyt R., Coffman R.L., O’Garra A. (2001). Interleukin-10 and the Interleukin-10 Receptor. Annu. Rev. Immunol..

[106] Alsayari A., Wahab S. (2021). Genus Ziziphus for the Treatment of Chronic Inflammatory Diseases. Saudi J. Biol. Sci..

[107] McCarthy J., O’Mahony L., O’Callaghan L., Sheil B., Vaughan E.E., Fitzsimons N., Fitzgibbon J., O’Sullivan G.C., Kiely B., Collins J.K. (2003). Double Blind, Placebo Controlled Trial of Two Probiotic Strains in Interleukin 10 Knockout Mice and Mechanistic Link with Cytokine Balance. Gut.

[108] Peña J.A., Rogers A.B., Ge Z., Ng V., Li S.Y., Fox J.G., Versalovic J. (2005). Probiotic Lactobacillus Spp. Diminish Helicobacter Hepaticus -Induced Inflammatory Bowel Disease in Interleukin-10-Deficient Mice. Infect. Immun..

[109] Karamese M., Aydin H., Sengul E., Gelen V., Sevim C., Ustek D., Karakus E. (2016). The Immunostimulatory Effect of Lactic Acid Bacteria in a Rat Model. Iran. J. Immunol..

[110] Borruel N. (2002). Increased Mucosal Tumour Necrosis Factor Alpha Production in Crohn’s Disease Can Be Downregulated Ex Vivo by Probiotic Bacteria. Gut.

[111] Reséndiz-Albor A.A., Reina-Garfias H., Rojas-Hernández S., Jarillo-Luna A., Rivera-Aguilar V., Miliar-García A., Campos-Rodríguez R. (2010). Regionalization of PIgR Expression in the Mucosa of Mouse Small Intestine. Immunol. Lett..

[112] Wahab S., Ahmad I., Irfan S., Baig M.H., Farouk A.-E., Dong J.-J. (2021). Use of Natural Compounds as a Potential Therapeutic Agent Against COVID-19. Curr. Pharm. Des..

[113] Ahmad M.D.F., Wahab S., Ali Ahmad F., Intakhab Alam M., Ather H., Siddiqua A., Amir Ashraf S., Abu Shaphe M., Idreesh Khan M., Ali Beg R. (2021). A Novel Perspective Approach to Explore Pros and Cons of Face Mask in Prevention the Spread of SARS-CoV-2 and Other Pathogens. Saudi Pharm. J..

[114] Ahmad M.F., Ahmad F.A., Khan M.I., Alsayegh A.A., Wahab S., Alam M.I., Ahmed F. (2021). Ganoderma Lucidum: A Potential Source to Surmount Viral Infections through β-Glucans Immunomodulatory and Triterpenoids Antiviral Properties. Int. J. Biol. Macromol..

[115] Yagi K., Huffnagle G.B., Lukacs N.W., Asai N. (2021). The Lung Microbiome during Health and Disease. Int. J. Mol. Sci..

[116] Philley J.V., Kannan A., Olusola P., Mcgaha P., Singh K.P., Samten B., Griffith D.E., Dasgupta S. (2019). Microbiome Diversity in Sputum of Nontuberculous Mycobacteria Infected Women with a History of Breast Cancer. Cell. Physiol. Biochem..

[117] Tunney M.M., Einarsson G.G., Wei L., Drain M., Klem E.R., Cardwell C., Ennis M., Boucher R.C., Wolfgang M.C., Elborn J.S. (2013). Lung Microbiota and Bacterial Abundance in Patients with Bronchiectasis When Clinically Stable and during Exacerbation. Am. J. Respir. Crit. Care Med..

[118] Hani U., Yasmin Begum M., Wahab S., Siddiqua A., Osmani R.A.M., Rahmathulla M. (2021). A Comprehensive Review of Current Perspectives on Novel Drug Delivery Systems and Approaches for Lung Cancer Management. J. Pharm. Innov..

[119] Claesson M.J., Cusack S., O’Sullivan O., Greene-Diniz R., de Weerd H., Flannery E., Marchesi J.R., Falush D., Dinan T., Fitzgerald G. (2011). Composition, Variability, and Temporal Stability of the Intestinal Microbiota of the Elderly. Proc. Natl. Acad. Sci. USA.

[120] Biagi E., Franceschi C., Rampelli S., Severgnini M., Ostan R., Turroni S., Consolandi C., Quercia S., Scurti M., Monti D. (2016). Gut Microbiota and Extreme Longevity. Curr. Biol..

[121] Rampelli S., Soverini M., D’Amico F., Barone M., Tavella T., Monti D., Capri M., Astolfi A., Brigidi P., Biagi E. (2020). Shotgun Metagenomics of Gut Microbiota in Humans with up to Extreme Longevity and the Increasing Role of Xenobiotic Degradation. mSystems.

[122] Wu L., Zeng T., Zinellu A., Rubino S., Kelvin D.J., Carru C. (2019). A Cross-Sectional Study of Compositional and Functional Profiles of Gut Microbiota in Sardinian Centenarians. mSystems.

[123] Mukhopadhya I., Segal J.P., Carding S.R., Hart A.L., Hold G.L. (2019). The Gut Virome: The ‘Missing Link’ between Gut Bacteria and Host Immunity?. Therap. Adv. Gastroenterol..

[124] Abt M.C., Osborne L.C., Monticelli L.A., Doering T.A., Alenghat T., Sonnenberg G.F., Paley M.A., Antenus M., Williams K.L., Erikson J. (2012). Commensal Bacteria Calibrate the Activation Threshold of Innate Antiviral Immunity. Immunity.

[125] Ichinohe T., Pang I.K., Kumamoto Y., Peaper D.R., Ho J.H., Murray T.S., Iwasaki A. (2011). Microbiota Regulates Immune Defense against Respiratory Tract Influenza A Virus Infection. Proc. Natl. Acad. Sci. USA.

[126] Taverniti V., Guglielmetti S. (2011). The Immunomodulatory Properties of Probiotic Microorganisms beyond Their Viability (Ghost Probiotics: Proposal of Paraprobiotic Concept). Genes Nutr..

[127] Gorbalenya A.E., Baker S.C., Baric R.S., de Groot R.J., Drosten C., Gulyaeva A.A., Haagmans B.L., Lauber C., Leontovich A.M., Neuman B.W. (2020). The Species Severe Acute Respiratory Syndrome-Related Coronavirus: Classifying 2019-NCoV and Naming It SARS-CoV-2. Nat. Microbiol..

[128] Zhu N., Zhang D., Wang W., Li X., Yang B., Song J., Zhao X., Huang B., Shi W., Lu R. (2020). A Novel Coronavirus from Patients with Pneumonia in China, 2019. N. Engl. J. Med..

[129] Wahab S., Ahmad I., Usmani S., Ahmad M.P. (2021). Efficacy of Dexamethasone for the Treatment of COVID-19 Infection: A Perspective Review. Curr. Drug Deliv..

[130] (2020). Long COVID: Let Patients Help Define Long-Lasting COVID Symptoms. Nature.

[131] Baradaran Ghavami S., Pourhamzeh M., Farmani M., Raftar S.K.A., Shahrokh S., Shpichka A., Asadzadeh Aghdaei H., Hakemi-Vala M., Hossein-khannazer N., Timashev P. (2021). Cross-Talk between Immune System and Microbiota in COVID-19. Expert Rev. Gastroenterol. Hepatol..

[132] Schuijt T.J., Lankelma J.M., Scicluna B.P., de Sousa e Melo F., Roelofs J.J.T.H., de Boer J.D., Hoogendijk A.J., de Beer R., de Vos A., Belzer C. (2016). The Gut Microbiota Plays a Protective Role in the Host Defence against Pneumococcal Pneumonia. Gut.

[133] Sencio V., Barthelemy A., Tavares L.P., Machado M.G., Soulard D., Cuinat C., Queiroz-Junior C.M., Noordine M.-L.L., Salomé-Desnoulez S., Deryuter L. (2020). Gut Dysbiosis during Influenza Contributes to Pulmonary Pneumococcal Superinfection through Altered Short-Chain Fatty Acid Production. Cell Rep..

[134] Keely S., Talley N.J., Hansbro P.M. (2012). Pulmonary-Intestinal Cross-Talk in Mucosal Inflammatory Disease. Mucosal Immunol..

[135] Yazar A., Atis S., Konca K., Pata C., Akbay E., Calikoglu M., Hafta A. (2001). Respiratory Symptoms and Pulmonary Functional Changes in Patients With Irritable Bowel Syndrome. Am. J. Gastroenterol..

[136] Morais A.H.A., Passos T.S., Maciel B.L.L., da Silva-Maia J.K. (2020). Can Probiotics and Diet Promote Beneficial Immune Modulation and Purine Control in Coronavirus Infection?. Nutrients.

[137] Jiang X., Hou X., Tang L., Jiang Y., Ma G., Li Y. (2016). A Phase Trial of the Oral Lactobacillus Casei Vaccine Polarizes Th2 Cell Immunity against Transmissible Gastroenteritis Coronavirus Infection. Appl. Microbiol. Biotechnol..

[138] Vabret N., Britton G.J., Gruber C., Hegde S., Kim J., Kuksin M., Levantovsky R., Malle L., Moreira A., Park M.D. (2020). Immunology of COVID-19: Current State of the Science. Immunity.

[139] Verdoni L., Mazza A., Gervasoni A., Martelli L., Ruggeri M., Ciuffreda M., Bonanomi E., D’Antiga L. (2020). An Outbreak of Severe Kawasaki-like Disease at the Italian Epicentre of the SARS-CoV-2 Epidemic: An Observational Cohort Study. Lancet.

[140] Anwar F., Altayb H.N., Al-Abbasi F.A., Al-Malki A.L., Kamal M.A., Kumar V. (2021). Antiviral Effects of Probiotic Metabolites on COVID-19. J. Biomol. Struct. Dyn..

[141] Isacco C.G., Ballini A., De Vito D., Nguyen K.C.D., Cantore S., Bottalico L., Quagliuolo L., Boccellino M., Di Domenico M., Santacroce L. (2020). Rebalancing the Oral Microbiota as an Efficient Tool in Endocrine, Metabolic and Immune Disorders. Endocr. Metab. Immune Disord. Drug Targets.

[142] Ballini A., Gnoni A., De Vito D., Dipalma G., Cantore S., Gargiulo Isacco C., Saini R., Santacroce L., Topi S., Scarano A. (2019). Effect of Probiotics on the Occurrence of Nutrition Absorption Capacities in Healthy Children: A Randomized Double-Blinded Placebo-Controlled Pilot Study. Eur. Rev. Med. Pharmacol. Sci.

[143] Fu Y.H., Wen J.B., Wang G.L., Wen P., Gong M., Han M., Li X. (2015). Effect of Enteral Nutrition on Cytokine Production and Plasma Endotoxin in Patients with Severe Acute Pancreatitis. World Chinese J. Dig..

[144] Kageyama Y., Nishizaki Y., Aida K., Yayama K., Ebisui T., Akiyama T., Nakamura T. (2021). Lactobacillus Plantarum Induces Innate Cytokine Responses That Potentially Provide a Protective Benefit against COVID-19: A Single-arm, Double-blind, Prospective Trial Combined with an in Vitro Cytokine Response Assay. Exp. Ther. Med..

[145] Martino H., Tonucci L., Santos K., Oliveira L., Ribeiro S. (2015). Effects of Probiotics on Glycemic Control and Inflammation in Type 2 Diabetes Mellitus: A Randomized, Double-Blind, Placebo-controlled Study. FASEB J..

[146] Xu K., Cai H., Shen Y., Ni Q., Chen Y., Hu S., Li J., Wang H., Yu L., Huang H. (2020). [Management of COVID-19: The Zhejiang Experience]. Zhejiang Da Xue Xue Bao Yi Xue Ban.

[147] Maruyama M., Abe R., Shimono T., Iwabuchi N., Abe F., Xiao J.-Z. (2016). The Effects of Non-Viable Lactobacillus on Immune Function in the Elderly: A Randomised, Double-Blind, Placebo-Controlled Study. Int. J. Food Sci. Nutr..

[148] Jespersen L., Tarnow I., Eskesen D., Morberg C.M., Michelsen B., Bügel S., Dragsted L.O., Rijkers G.T., Calder P.C. (2015). Effect of Lactobacillus Paracasei Subsp. Paracasei, L. Casei 431 on Immune Response to Influenza Vaccination and Upper Respiratory Tract Infections in Healthy Adult Volunteers: A Randomized, Double-Blind, Placebo-Controlled, Parallel-Group Study. Am. J. Clin. Nutr..

[149] Van Puyenbroeck K., Hens N., Coenen S., Michiels B., Beunckens C., Molenberghs G., Van Royen P., Verhoeven V. (2012). Efficacy of Daily Intake of Lactobacillus Casei Shirota on Respiratory Symptoms and Influenza Vaccination Immune Response: A Randomized, Double-Blind, Placebo-Controlled Trial in Healthy Elderly Nursing Home Residents. Am. J. Clin. Nutr..

[150] Boge T., Rémigy M., Vaudaine S., Tanguy J., Bourdet-Sicard R., van der Werf S. (2009). A Probiotic Fermented Dairy Drink Improves Antibody Response to Influenza Vaccination in the Elderly in Two Randomised Controlled Trials. Vaccine.

[151] Mullié C., Yazourh A., Thibault H., Odou M.F., Singer E., Kalach N., Kremp O., Romond M.B. (2004). Increased Poliovirus-Specific Intestinal Antibody Response Coincides with Promotion of Bifidobacterium Longum-Infantis and Bifidobacterium Breve in Infants: A Randomized, Double-Blind, Placebo-Controlled Trial. Pediatr. Res..

[152] Kukkonen K., Nieminen T., Poussa T., Savilahti E., Kuitunen M. (2006). Effect of Probiotics on Vaccine Antibody Responses in Infancy—A Randomized Placebo-Controlled Double-Blind Trial. Pediatr. Allergy Immunol..

[153] Bosch M., Méndez M., Pérez M., Farran A., Fuentes M.C., Cuñé J. (2012). Lactobacillus Plantarum CECT7315 and CECT7316 Stimulate Immunoglobulin Production after Influenza Vaccination in Elderly. Nutr. Hosp..

[154] Rizzardini G., Eskesen D., Calder P.C., Capetti A., Jespersen L., Clerici M. (2012). Evaluation of the Immune Benefits of Two Probiotic Strains Bifidobacterium Animalis Ssp. Lactis, BB-12^®^ and *Lactobacillus paracasei* Ssp. Paracasei, L. Casei 431^®^ in an Influenza Vaccination Model: A Randomised, Double-Blind, Placebo-Controlled Study. Br. J. Nutr..

[155] Akatsu H., Arakawa K., Yamamoto T., Kanematsu T., Matsukawa N., Ohara H., Maruyama M. (2013). Lactobacillus in Jelly Enhances the Effect of Influenza Vaccination in Elderly Individuals. J. Am. Geriatr. Soc..

[156] Van Landingham C.B., Keast D.R., Longnecker M.P. (2021). Serum Concentration of Antibodies to Mumps, but Not Measles, Rubella, or Varicella, Is Associated with Intake of Dietary Fiber in the NHANES, 1999–2004. Nutrients.

[157] Scott K.P., Grimaldi R., Cunningham M., Sarbini S.R., Wijeyesekera A., Tang M.L.K., Lee J.C.-Y., Yau Y.F., Ansell J., Theis S. (2020). Developments in Understanding and Applying Prebiotics in Research and Practice—An ISAPP Conference Paper. J. Appl. Microbiol..

[158] de Jong S.E., Olin A., Pulendran B. (2020). The Impact of the Microbiome on Immunity to Vaccination in Humans. Cell Host Microbe.

[159] Van Hoffen E., Ruiter B., Faber J., M’Rabet L., Knol E.F., Stahl B., Arslanoglu S., Moro G., Boehm G., Garssen J. (2009). A Specific Mixture of Short-Chain Galacto-Oligosaccharides and Long-Chain Fructo-Oligosaccharides Induces a Beneficial Immunoglobulin Profile in Infants at High Risk for Allergy. Allergy Eur. J. Allergy Clin. Immunol..

[160] Schouten B., Van Esch B.C.A.M., Hofman G.A., Boon L., Knippels L.M.J., Willemsen L.E.M., Garssen J. (2010). Oligosaccharide-Induced Whey-Specific CD25+ Regulatory T-Cells Are Involved in the Suppression of Cow Milk Allergy in Mice. J. Nutr..

[161] Vos A.P., Haarman M., VanGinkel J.W.H., Knol J., Garssen J., Stahl B., Boehm G., M’Rabet L. (2007). Dietary Supplementation of Neutral and Acidic Oligosaccharides Enhances Th1-Dependent Vaccination Responses in Mice. Pediatr. Allergy Immunol..

[162] van’t Land B., Schijf M., van Esch B.C.A.M., van Bergenhenegouwen J., Bastiaans J., Schouten B., Boon L., Garssen J. (2010). Regulatory T-Cells Have a Prominent Role in the Immune Modulated Vaccine Response by Specific Oligosaccharides. Vaccine.

[163] Gill H., Prasad J. (2008). Probiotics, Immunomodulation, and Health Benefits. Adv. Exp. Med. Biol..

[164] Davidson L.E., Fiorino A.-M., Snydman D.R., Hibberd P.L. (2011). Lactobacillus GG as an Immune Adjuvant for Live-Attenuated Influenza Vaccine in Healthy Adults: A Randomized Double-Blind Placebo-Controlled Trial. Eur. J. Clin. Nutr..

[165] Torp A.M., Bahl M.I., Boisen A., Licht T.R. (2022). Optimizing Oral Delivery of next Generation Probiotics. Trends Food Sci. Technol..

[166] Derrien M., van Hylckama Vlieg J.E.T. (2015). Fate, Activity, and Impact of Ingested Bacteria within the Human Gut Microbiota. Trends Microbiol..

[167] O’Toole P.W., Marchesi J.R., Hill C. (2017). Next-Generation Probiotics: The Spectrum from Probiotics to Live Biotherapeutics. Nat. Microbiol..

[168] Derrien M., Vaughan E.E., Plugge C.M., de Vos W.M. (2004). Akkermansia Municiphila Gen. Nov., Sp. Nov., a Human Intestinal Mucin-Degrading Bacterium. Int. J. Syst. Evol. Microbiol..

[169] Foditsch C., Santos T.M.A., Teixeira A.G.V., Pereira R.V.V., Dias J.M., Gaeta N., Bicalho R.C. (2014). Isolation and Characterization of Faecalibacterium Prausnitzii from Calves and Piglets. PLoS ONE.

[170] Laursen M.F., Laursen R.P., Larnkjær A., Michaelsen K.F., Bahl M.I., Licht T.R. (2017). Administration of Two Probiotic Strains during Early Childhood Does Not Affect the Endogenous Gut Microbiota Composition despite Probiotic Proliferation. BMC Microbiol..

[171] Kristensen N.B., Bryrup T., Allin K.H., Nielsen T., Hansen T.H., Pedersen O. (2016). Alterations in Fecal Microbiota Composition by Probiotic Supplementation in Healthy Adults: A Systematic Review of Randomized Controlled Trials. Genome Med..

[172] Minelli E.B., Benini A. (2008). Relationship between Number of Bacteria and Their Probiotic Effects. Microb. Ecol. Health Dis..

[173] Fenster K., Freeburg B., Hollard C., Wong C., Laursen R.R., Ouwehand A.C. (2019). The Production and Delivery of Probiotics: A Review of a Practical Approach. Microorganisms.

